# Evaluation of Triple Fragment Vaccine HSPX (*Rv2031c)* + PPE44 (*Rv2770c*) + Mouse IgG_1 _(*Fcγ2a) *with Auxiliary Adjuncts IL-22 in Comparison with BCG Vaccine

**DOI:** 10.30699/IJP.2022.549673.2849

**Published:** 2022-08-13

**Authors:** Azar Valizadeh, Afra Khosravi, Hamid Sedighian, Elham Behzadi, Elaheh Gholami Parizad, Abbas Ali Imani Fooladi

**Affiliations:** 1 Clinical Microbiology Research Center, Ilam University of Medical Sciences, Ilam, Iran; 2 Applied Microbiology Research Center, Systems Biology and Poisonings Institute, Baqiyatallah University of Medical Sciences, Tehran, Iran; 3 Academy of Medical Sciences of the I. R. of Iran, Tehran, Iran

**Keywords:** HSPX, PPE44, IL-22, Subunit

## Abstract

**Background & Objective:**

Despite the vaccination with the BCG vaccine, tuberculosis (TB) remains one of the major health problems in the world. The aim of this study was to evaluate our newly designed vaccine using IL-22 as an adjuvant in comparison with the common BCG vaccine.

**Methods:**

The gene constructs were cloned into the expression vector of pET28a and then into the recombinant vector of PET28a – HSPX, and PPE44 was transformed into *Escherichia coli* BL21 (DE3). Finally, the immunogenicity of recombinant proteins with and without BCG and IL-22 in BALB/c mice was investigated.

**Results:**

The key cytokines INF-γ and TNF-α were elevated more greatly in BCG immunized group than in PHF immunized group.

Immunization with PHF showed a significant increase in IL-4 levels versus the BCG group. Adding IL-22 to the vaccine formulations indicated a tiny increase in IL-4 levels compared to their related vaccine groups.

Specific total IgG1 in the experimental groups showed an increase in comparison with control groups, but in the vaccinated groups, no significant differences were observed, and the presence of IL-22 in the vaccine formulations indicated a slight decrease compared with the related mere vaccine groups. Results of specific total IgG2a in the experimental groups revealed that only in the PHF group formulated with IL-22 a significant increase occurs compared with all other experimental groups.

**Conclusion:**

It seems that BCG, as the only licensed vaccine for TB infection, could be more potent than a recombinant vaccine in the induction of cellular and humoral immune responses.

## Introduction

The *Mycobacterium tuberculosis* (*M. tuberculosis*) is the causative agent of TB in humans (1). As a pervasive threat to communities, *M. tuberculosis* was discovered in March 1882 by Robert Koch, who subsequently received the Nobel Prize. Tuberculosis (TB) is considered a "human plague"(2). One-third of the world population is supposed to become infected with *M. tuberculosis* in the 21^st^ century, according to the world health organization (WHO). Each year, a million and seven hundred thousand patients die of this disease (1, 3). TB associated with HIV has increased the mortality of patients, which may occur in a variety of forms, including respiratory infections that contain the bacteria from an infected person depending on some factors such as the incubation period, duration of the infectiousness (4), the distance from the source of the infection, the extent and the rate of the infection and extensively drug-resistant TB (XDR-TB) strains (1). Global control of TB has many problems, including difficulty in detecting the illness timely, lack of an effective vaccine, simultaneous TB infection, long-term chemotherapy, and the resistance to MDR (Multidrug-resistant( and XDR (Extensively drug-resistant). Therefore, designing and producing a new vaccine is necessary to reduce the 8-10 million new TB infections each year (4, 5). In 2012, 6.8 million new cases of infection had occurred, and over 1.3 million deaths were registered (6). Now, around one-third of the world population is contaminated with TB, and about two billion people have latent TB infection, of which 5 to 10 percent of those infected will develop active TB (7). 

Given the issue's importance, the WHO aimed to reduce the incidence of TB by 50% since 2015 and eradicate infection by 2050. According to research reports from 22 countries, about 80% of the complications and cases of TB-related deaths are reported from countries such as India, China, South Africa, Nigeria, and Indonesia, ranked first to fifth. China, India, Russia, South Africa, and Bangladesh have the most cases of drug-resistant TB (2).

The protective effect of Bacilli Calmette-Guérin (BCG) was detected in 1921 when two anti-TB antibodies derived from *Mycobacterium bovis *(*M. bovis*) were introduced by two scientists named Calmette and Guérin. The findings showed that the BCG vaccine had a drawback, including its variable protective effect against pulmonary TB in adults, i.e., its protective spectrum varies from 0 to 80%, and its sustained effect is 6 to 12 months after the vaccination (8).

On the other hand, the other deficiency of the BCG vaccine is that it can protect neonates only against tuberculous meningitis (TBM). Various studies and articles have pointed out other factors that are of importance in the effectiveness of the BCG vaccine, including the difference in protection against pulmonary TB, reduced immunological memory, environmental contamination with *mycobacterium* (especially in tropical and temperate regions), genetic variation (both in hosts and in BCG strains) (9). Previous studies have shown that re-vaccination and booster injections are not so effective in preventing the onset of bruising in adults (10). Therefore, in Southern India, it was suggested that BCG should be avoided (4).

Thus, for BCG production, it is necessary to achieve three goals as follows: 1) Providing a fast-diagnostic approach, 2) Considering the cost-effectiveness 3) Having more effect than the existing vaccine (4, 5). So far, various types of vaccines have been developed to create immunity against *M. tuberculosis*, including subunit vaccines, recombinant vaccines, live, attenuated vaccines, and DNA vaccines (11). Nowadays, more developed vaccines are DNA and recombinant vaccines because they create sustained and long-term secure responses and are safer than live, attenuated vaccines (especially in patients with AIDS); for their production and maintenance, there is no need to create a cold chain, and it has been observed that immunization with a large number of recombinant DNA vaccines including a plasmid containing the gene ESAT-6 (The 6 kDa early secretory antigenic) and IL-12 N220Land Ag85A (Secreted antigen Ag85A ) and HSP results in a decrease in bacterial levels in the mouse model (11, 12).

In recent years, these vaccines have become more frequent in humans. New approaches are employed to increase the immunogenicity of these vaccines in humans, including various genetic adjuvants (e.g., cloning of genes encoding cytokines with genes encoding antigens), electroporation, and the prime-boost method. Clearly, the results of these methods have shown an increase in the immune response of Th1 and TCD8 cells (13).

Antigens such as HSP60, HSP70, ESAT-6, PPE44, and HSPX are new candidates for the vaccination against TB or are used as diagnostic agents (14), which recruit multiple antigens to have stronger safety (15). Many researchers and health workers who have the potential to completely eradicate TB with an effective vaccine, especially in developing countries where TB is higher, are faced with a shortage of financial resources to access the treatment. Therefore, the need for a highly effective vaccine is of great importance. Over the past two decades, the research budget for the TB vaccine development has quadrupled and reached more than half a billion dollars. This budget is estimated to be more than four times the money required to achieve effective vaccine production against TB (10, 16). According to estimates provided by WHO from 2006 to 2015, it took about $ 60 billion to eradicate TB. In 2010, $ 2.64 billion was funded to control this disease in 22 countries (2).

The genome of *M. tuberculosis* contains 4999 genes, and about 3.199 proteins are isolated from the supernatant of the bacterial culture (8). Recently, the researchers have been investigated to obtain the new antigens for the vaccine against TB. Here, we have tried to evaluate the effectiveness of the vaccine. In this research, a new recombinant vaccine was assessed alone and compared to BCG.

## Material and Methods

**Design of the Construct and Gene Optimization**

According to the *in silico* studies, the sequences of HSPX, PPE44, and FC genes based on the genome of the *M. tuberculosis* strain (H37RV) were obtained from the NCBI reference sequences (Ref Seq) database, and the annotation of the genome was retrieved from GenBank AL123456.3 and 444893469: 4380453-4381940 for the PPE44 gene and GenBank M76712.1 | MSG14KA: 16-450 for the HSPX gene and GenBank V00798.1, 699 bp for the FC gene. The activated amino acid sequences were also downloaded from the UniPort with access number P9WHZ3 for PPE44 and P9WMK1 for the HSPX protein and FASTA format. To maintain the structure of the recombinant protein and avoid interference, the common domains included a repeater linker between HSPX and PPE44 consisting of 17 amino acids "AEAAAKEAAAKEAAAKA" and a subsequent linking sequence between PPE44 and FC for the construct, including "GGGGSGGGGS" were selected. Due to the presence of salt bridges, these linkers can prevent the closure of a stable helix structure. Moreover, the analysis and prediction of the three-dimensional (3D) structure of the models were performed using the ITASSER server. The second structure of the protein was predicted using 2D-structure web servers. Coiled-coil and single-alpha-helix prediction for protein structure, GC percentage, and codon optimization were performed using Java Causal Analysis Toolkit software (JCat) (http://www.jcat.de/).

The spin software (http://www.ncbi.nlm.nih.gov/gorf/gorf.html) was also used to obtain the optimum coding sequence and thus improve the expression of the recombinant proteins named valizadeh1.

The analysis of protein sequences with the CLC software and the ExPASy ProtParam online server included factors such as hydrophobicity, solubility, environmental stability, molecular weight, and also a prediction of the overall antigenicity of the proteins were performed in the VaxiJen (vaccine design) v2.0 server online web server (threshold 0.4%) using a designed construct in pET28a expression vector with a restriction site. Restriction enzyme cutting sites were analyzed using online webcutter version 2.0 (http://rna.lundberg.gu.se/cutter2/) and Nebcutter version 1.0 servers (http://tools.neb.com/NEBcutter). In CLC genomics workbench version 7.0 (Qiagen, Redwood City), the desired gene was introduced into the expression vector using BamHI and XhoI.

**Determination of 3D Structure of the Fusion Protein PPE44 + HSPX + Fc**

Twenty models were developed by using the Modeler program (https://salilab.org/modeller/). Then, one of the models with the highest ERRAT score was chosen for structural changes. The final model had an ERRAT score of 90%. The Ramachandran showed that ˃ 98% of the fusion protein fell under the plot's most favorable and allowed regions.

**Construction of Chimeric Gene**

The designed gene was synthesized by Biomatik Company (Biomatik, Ontario, Canada), and the optimized gene was cloned in the pET28a vector. 

**Transformation and Expression of Recombinant Protein**

The recombinant HSPX-PPE44-Fc vector was transferred into *Escherichia coli* BL21 (DE3) expression host. Transformed bacteria were cultured in 5 mL LB (Luria-Bertani) medium containing 20 mg/mL kanamycin and incubated at 37°C overnight. When OD600 reached 0.6-0.8, the bacterial cultures were induced by 1 mM isopropyl-beta-D-thiogalactopyranoside (IPTG).

Subsequently, the cultures were incubated again at 37°C overnight. Then, the expression of HSPX- PPE44-Fc was evaluated by sodium dodecyl sulfate-polyacrylamide gel electrophoresis (SDS/PAGE). The different concentrations of IPTG (0.5, 0.6, 0.7, 0.8, 0.9, and 1 mM) with various time durations (2, 4, 6, 8 h) were examined to optimize protein expression.

**Isolation and Purification of Chimeric Recombinant Proteins**

The bacteria were gathered 16 h after IPTG treatment to isolate the recombinant protein. The pellets were washed twice with phosphate-buffered saline (PBS) (0.1 m, pH: 7.4). The bacterial pellets were (4 g) resuspended in 20 mL of lysis buffer (50 mM Tris–HCl containing 1mM EDTA, 5 mM DTT), and the cells were disrupted by sonication five times for 30 S at 200 W. The resulting solutions were centrifuged at 6000×g at 4°C for 30 min. The supernatant was discarded and the pellets were washed twice with washing buffer (1 M urea in 50 mM Tris–HCl, pH: 8.0, containing 5 mM EDTA and 1 mM DTT), then centrifuged at 11000×g at 25°C for 15 min. To extract proteins from washed inclusion bodies, urea buffer (0.01M Tris/base, 0.1 M NaH_2_PO_4_, 8M Urea, pH: 8) was used. After incubation at room temperature for 16 h, the washed pellets were centrifuged for 20 min, and the supernatant was analyzed by SDS/PAGE. Subsequently, the supernatant that contained recombinant fusion proteins was loaded onto Ni-NTA affinity column (QIAGEN, Germany). The column was washed with washing buffer (50 mm NaH_2_PO_4_, 20 mm imidazole, 300 mm NaCl, pH: 8), and unbound proteins were removed. Recombinant proteins attached to the column were eluted with elution buffer (50 mM NaH2PO4, 250 mM imidazole, 300 mM NaCl pH: 8). Dialysis was performed to remove imidazole. 

**Western Blot Analysis**

The expressed recombinant protein from the polyacrylamide gel was transferred to polyvinylidene difluoride (PVDF) paper in semi-arid conditions (Bio-Rad). The PVDF membrane was blocked with 3% skim milk solution in 0.1 mol/L PBS containing 0.05% Tween 20 (PBS-T) at 4°Ċ overnight. In the next step, after washing the paper with PBST, the membrane was incubated with horseradish peroxidase (HRP)-conjugated mice anti-His-tag antibody (diluted 1:2000 in PBS-T) (Sigma, USA) at room temperature for 45 min while shaking. After washing by PBST, the membrane was stained with dye di-amino-benzidine (DAB, Sigma USA).

**Preparation and Immunogenicity of Subunit Vaccine**

After desalinization with Vivaspin20 columns (Sartorius Stedim, Germany) and also LPS detoxification using the ToxinEraser^TM^ Endotoxin Removal Kit (GenScript, USA), the HSPX- PPE44 -FC fusion protein in PBS (0.2 mg/mL) was resolved. The Balb/C mice (6-8 weeks) and adjuvant IL-22 were purchased from Pasteur Institute of Iran (Tehran, Iran) and InvivoGen (InvivoGen Company, USA), respectively. The subcutaneous injection was performed at a final volume of 1 mL (0.2 mg/mL). The mice were randomly divided into five groups (n=7 per group) including (i) the recombinant HSPX - PPE44 -FC protein without adjuvant (HPF) treated group (ii) the group that received the recombinant protein HSPX- PPE44 -FC plus the IL-22 adjuvant (HPF-IL22) (iii) the group that treated with the recombinant protein HSPX- PPE44 -FC +BCG (iv) the recombinant protein HSPX- PPE44 -FC +BCG and IL-22 adjuvant (HPF-BCG-IL22) treated group (v) the group that received the BCG vaccine ( BCG) and (vi) a control group treated with PBS. 

The vaccines were injected once on day 0 and the second on day 21. Blood samples were collected on days 14 and 45 after the first injection (100 µL protein HSPX- PPE44 -FC, 50 µL of adjuvant IL-22, 50 µL of adjuvant BCG). 

Ultimately, the mice were sacrificed 25 days after the final immunization (46 days after the first injection). The vaccinated mice and the control group's spleen were isolated in an aseptic condition and homogenized by a cell strainer. A cell suspension was prepared, and the erythrocyte cells were removed by a leaching buffer (ammonium chloride). 10 × 10^6 ^cells/m^2^ of extracted spleen cells were added to the wells in duplicates (12 wells plates) containing 500 μL of RPMI1640 medium - plus Penicillin/Streptomycin (Penstrep, Biosera, UK) and 10% of the fetal bovine serum (FBS) (GIBCO, UK). Spleen cells in microplate were divided into two treatment groups (i) 10 μL of HSPX- PPE44 -FC protein and (ii) 5 μg/mL of IL-22. After 72 hours of incubation at 37°C, the culture medium supernatant was collected. The level of Interferon-gamma IFN-γ, IL-2, IL-4, IL-10 TNF-α and TFG were also measured based on IgG1, IgG2a found in the supernatant by ELISA kit (eBioscience, Austria) according to the manufacturer's instructions 

**Statistical Analysis**

Results are presented as mean ± SEM. The results were analyzed by one-way ANOVA using SPSS version 16 (SPSS Inc., Chicago, IL., USA). Statistical significance was determined at P-value≤0.05. The graphs were carried out using the GraphPad Prism 6 (GraphPad Software Inc., La Jolla, San Jose, CA, USA)**.**

## Results

**Molecular Modeling of HSPX- PPE44-Fc Recombinant Protein**

**Fig. 1:**
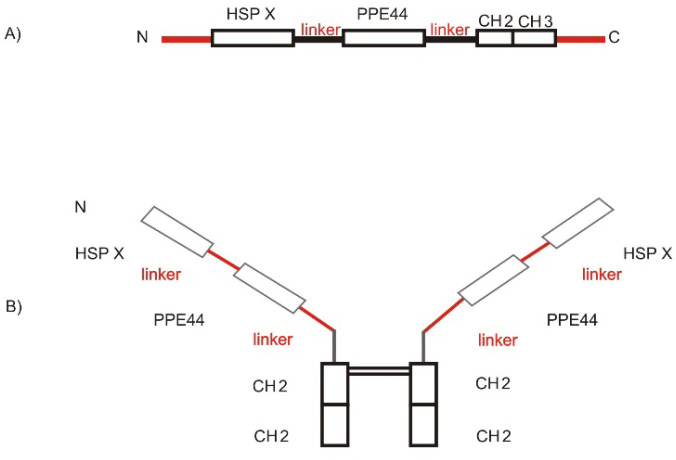
The schematic shape of the recombinant protein construction. Fig. A: Schematic shape of the recombinant protein HSPX- PPE44-Fc Fig. B: Schematic shape of the dimer form of Fc recombinant protein

At first, more than 20 models of the protein were selected by modeling software (https://salilab.org/modeller/), and one of them with the best ERRAT score was selected for structure modeling and refinement. The disulfide bonds were added to the chain structure during the process of protein modeling in the form of a dimer. Optimization of the mechanical geometry of the molecule in the form of a dimer structure for the protein is shown in Figure 1.

**Final Solvent Surface and Solid Ribbon Structure**

**Fig. 2:**
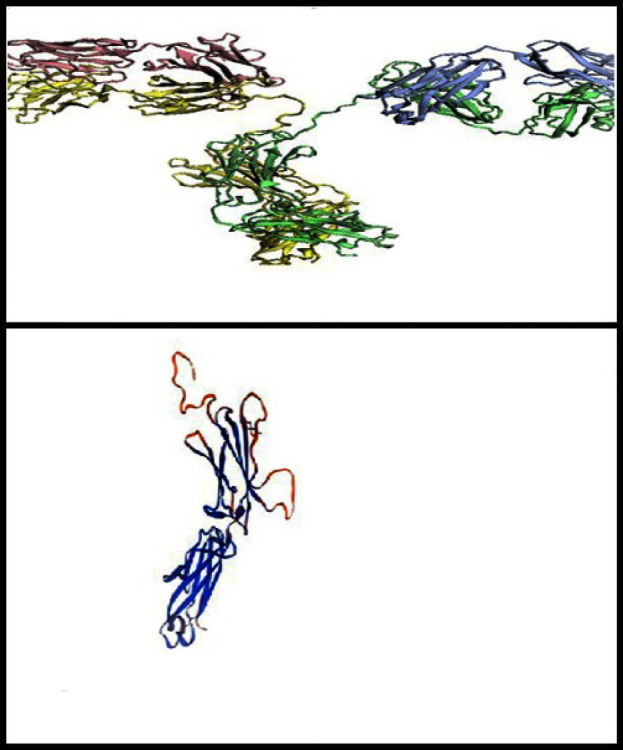
Model-based on SWISS-MODEL software

**Fig. 3:**
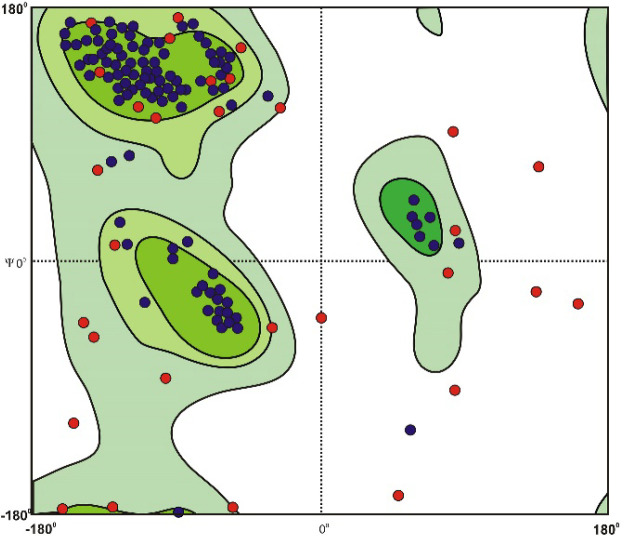
The Ramachandran Chart

The final model is shown in Figures 2 and 3. The final model has an ERRAT score of 97%, which confirms by the Ramachandran chart as an acceptable structure with a validity of over 99%.

**Optimization of Recombinant Protein Production**

**Fig. 4:**
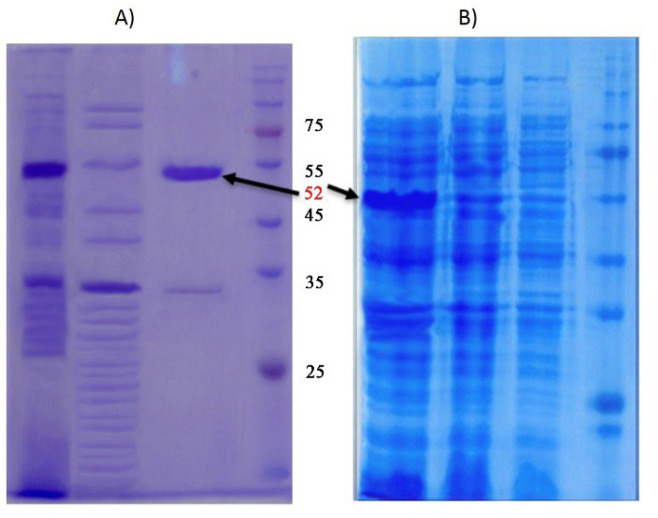
In the SDS-PAGE analysis, the recombinant protein HSPX- PPE44-Fc purified has a moleculecular weight band 52 KDa

To find the best environmental conditions to achieve the highest protein production, the fusion protein HSPX- PPE44-Fc was designed under varied conditions such as different concentrations of IPTG, distinct temperatures and various incubation times. Finally, the best SDS-PAGE analysis and Western blot at 0.9 mM IPTG at 37°C overnight was the best condition for achieving the highest recombinant protein content. The recombinant fusion protein was expressed using a 6-His-tag at N-terminus to facilitate the purification by nickel–nitrilotriacetic acid (Ni-NTA) resin column (Bioneer, Daejeon, South Korea) (Figure 4a, 4b).

**The Immune Response Pattern**

The IL-4, IFN-γ, and TNF-α cytokines (n=7 per group) released from spleen cells into the supernatant were measured by ELISA. Evaluation of immune responses against recombinant proteins, including IL-4, IFN-γ, and IL-4, as well as IgG1 and IgG2a, were assessed after vaccination on day 45 by culturing the spleen cells.

**Th1 (INF-γ) and TNF-α Responses**

**Fig. 5:**
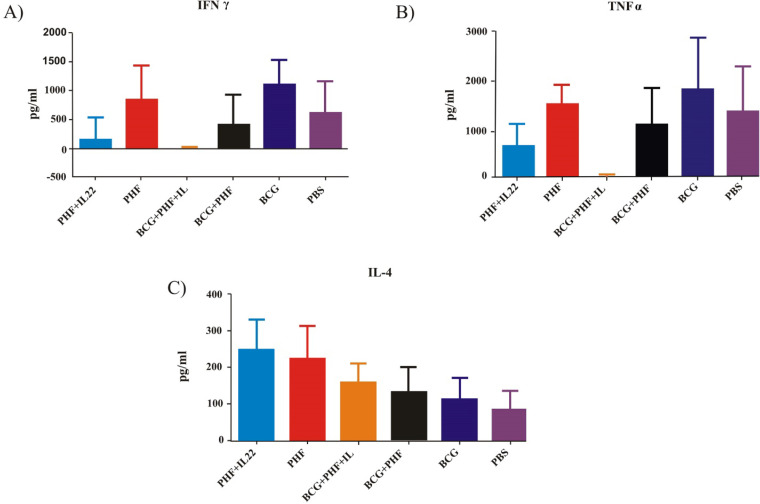
**Th1 (INF-γ), TNF-α, and IL-4 responses**. **A:** The cytokine profile of INF-γ in different experimental groups receiving from different vaccine formulations **B:** TNF-α release in the different experimental groups **C:** IL-4 response in the different experimental groups

The best response to the INF-γ cytokine was seen in the BCG immunized group, which was 30.37% higher than the PHF immunized group. Also, the formulation of vaccines with IL-22 does not positively affect the INF-γ cytokine release. The response seems suppressed compared to the related mere vaccine group (Figure 5A).

In addition, the highest TNF-α level was observed in the BCG immunized group, which was about 23% higher than PHF immunized group. Furthermore, the addition of IL-22 in the vaccine formulation was suppressed in PHF and BCG vaccines compared to mere vaccine groups (Figure 5B). 

**Th2 (IL4) Responses**

Immunization with PHF in Figure 5C shows a significant increase in IL-4 levels compared to the BCG group (*P*=0.0203). The addition of IL-22 to the vaccine formulations shows a tiny increase in IL-4 levels compared to their related vaccine groups.

**Response Level of IgG1 and IgG2A**

**Fig. 6:**
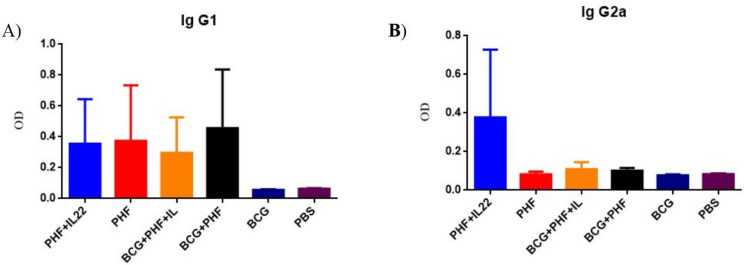
**IgG1 and IgG2A responses. A:** IgG1 response in the different experimental groups receiving from different vaccine formulations. **B:** IgG2A response n the different experimental groups

Specific total IgG1 in the experimental groups (Figure 6A) shows an increase versus control groups, but in the vaccinated groups, no significant differences were observed, and the presence of IL-22 in the vaccine formulations showed a tiny decrease in comparison to their related mere vaccine groups.

Results of specific IgG2a in the experimental groups show that only the PHF group formulated with IL-22 showed a significant increase compared to all other experimental groups (Figure 6B).

**Level of Immune Responses**

**Fig. 7:**
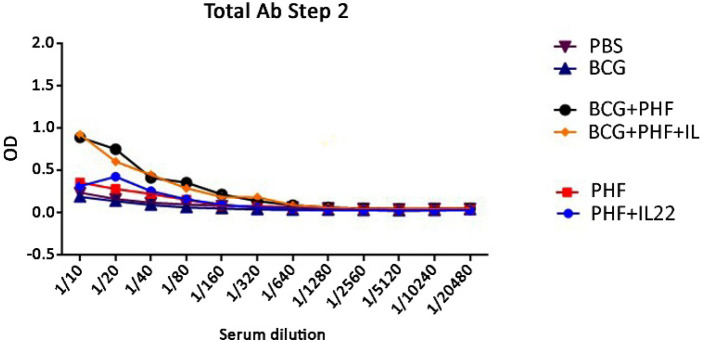
Assessment of the specific total IgG response on day 45 after the final shooting. The results show that BCG was more potent than the PHF group in the induction of humoral immune responses, and IL-22 in the vaccine formulation does not show a positive effect in the induction of humoral immune response in the PHF and BCG+PHF groups as compared with mere vaccines

Twelve vaccine dilutions were evaluated on days 14 and 45 after injection to determine the dose and dilution rate. The amount of these dilutions was used to determine the best injection dose compared with the control group only receiving BCG. According to Figure 7, the results are significant or not against BCG routine vaccine.

## Discussion

While Bacillus Calmette-Guérin (BCG) is the only approved vaccine against tuberculosis infection, several vaccine candidates are under study to achieve a more potent agent. It is clear that BCG is not potent in various countries, and an alternative vaccine with a higher degree of potency is needed. Several immunogens derived from the structure of the bacteria have been shown to protect the animal models against this infectious disease, but none of them has been approved to be applied as a routine vaccine to induce protection in human beings. It is clear that targeting more antigens at the bacterial cell structure could induce more potent immune responses that increase the chance of protection. Besides this strategy, utilizing a potent adjuvant could induce protection by increasing the immunogenicity of antigen and modulation of immune responses toward a desired pattern needed for the elimination of pathogens.

Herein, we used PPE44: HSPX: FC complex (PHF) as a novel vaccine, and to increase the vaccine potency, IL-22 is used as an adjuvant. PPE44 protein rich in T cell epitope and as immunogen induces cellular immune responses is identified as a novel antigen of *M. tuberculosis,* and its role as the subunit vaccine is comparable to the protectivity of BCG that is reported previously (17). In addition, studies show the positive effect of the fusion HSPX vaccine against *M. tuberculosis* has a higher protectivity level (18). Furthermore, to increase the vaccine immunogenicity, these candidate molecules were fused with Fc domain of immunoglobulin to target this vaccine to the Fc receptors on the surface of dendritic cells, thereby providing strong signals for T cells activation (19). In this study, to boost the responses, IL-22 cytokine is used in the vaccine formulation to increase the vaccine immunogenicity, as reported by other researchers on the various vaccine models (20-22).

The immunoassay results in the experimental groups demonstrated that mice immunized with BCG showed the highest IFN-γ cytokine response and 30.37% higher than the PHF immunized group. In addition, the utilization of IL-22 as an adjuvant in the PHF and BCG+PHF vaccine formulation suppressed the induction of IFN-γ compared to their related mere vaccine groups.

INF-γ is one of the most important immunological factors contributing to the polarization and activation of the Th1 response as the most important arm of the immune response against TB. In fact, INF-γ released by Th1 cells triggers the activation of macrophages, and activated macrophages kill the bacteria by releasing nitric oxide and other immunologic products (21-23). 

A study by Kaur* et al.* showed the positive effect of rIL-22 as an adjuvant against *Salmonella typhi* and *Salmonella Typhimurium* to induce the Th1 response (20). In the other study on the antigen of the *lack* gene of *Leishmania major*, it has been shown that IL-22 as an adjuvant increased the IFN-γ cytokine response and the Th1 pattern formation (22). These studies proved the adjuvant effect of IL-22 in combination with the vaccine to induce the IFN-γ cytokine response, while in the TB vaccine model, we didn't achieve such a result here, and this controversy may be due to the type of antigen or doses of IL-22 in this study.

Activated Th2 by producing IL-4 and IL-10 cytokines suppresses Th1 responses, contributes to the inflammation induced through this pathway, and plays an important role in stimulating antibody production. However, they can help replicate TB elsewhere (20, 22-24). According to the other findings, PPE44 and HSPX subunits can differentiate naive Th cells into Th1 and induce INF-γ production, which is a strong and effective response to the intracellular bacteria. The same naive T cells can be isolated in the presence of cyanides such as IL-1B to Th2 and produce cytokines such as IL-4 and IL-10 to induce a protective response against the extracellular bacteria. The type of Cayo Cayne is dependent on the stimulation caused by the antigenic index (20, 22, 24, 25).

In another study, the simultaneous use of BCG and plasmid coding PPE44 increased the T-cell and B-cell responses in C57BL/6 mice more than when BCG was administrated alone (26). Therefore, collaborating with the primary enhancer will increase the effectiveness of the DNA vaccine in stimulating immune responses.

These findings suggest that PPE44 is responsible for the immune response against TB and can be used as a DNA vaccine against it (27). PPE44 antigen is a bacterial virulence factor obtained from *M. tuberculosis* and is one of the protein family of PPE (Pro-Pro-Glu) N-terminal. In various studies, PPE44 has been introduced as a new and suitable candidate for the vaccine and is produced in the pathogenic strains of the Beijing Brigade more than the other strains. The immune response produced against this antigen in the rat can be detected early and delayed. This antigen has distinct epitopes that can be detected exclusively by MHCI and MHCII and can be adequately protective if used as a subunit vaccine or as a DNA vaccine (13, 25).

It is logical to use antigens and design an effective vaccine against TB. A proper vaccine contains HSPX antigens that are proven to protect mice against the aerosols of *M. tuberculosis* (28). 

HSPX is an immune-stimulating antigen that can result in host immunity against primary TB and secondary activated infection. The first gene induced by TB bacteria under low oxygen and latency conditions is HSPX (16-kDa). This crystalline-α protein is also said to be a type of heat shock protein and is present in bacteria that help the bacteria to survive in macrophages. The production of this protein increases in the stationary phase of growth (29, 30).

Recent research has shown that the level of HSPX- associated IFN-γ in patients was higher than in the BCG- vaccinated individuals, indicating this antigen's importance in the pathogenesis of *M. tuberculosis*. 

In addition, as this antigen is produced both in the latency phase of infection and at the end of the logarithmic phase of bacterial growth, it has been considered a candidate for a novel vaccine that can be used in all phases of infection. The association of T and B cells was detected in the studies conducted on the patients with active immune responses (31).

Considering the efficacy of the BCG vaccine in children and the booster effect and immunogenicity of antigens such as HSPX in combination with the BCG vaccine, it is possible to reduce the risk of a highly infectious TB by designing and manufacturing the hybrid vaccines in epidemic areas (28).

Several recent studies have been conducted on the immune response by administration of the HSPX vaccine. Groupie* et al.* used HSPX to design and produce a new fusion vaccine, similar to our study (7). Yuan* et al.* reported high levels of IFN-γ and IL-2 after injection and obtained a strong cellular immune response against *M. tuberculosis*. These findings indicate that proteins expressed in the latent phase of the TB infection are as potent as antigens produced by *M. tuberculosis* (7). 

Moreover, a high level of INF-γ was obtained when the BCG vaccine was used as a prime-boost or as the subunit vaccine and adjuvant and was significantly associated with the group receiving only the BCG vaccine. Studies proved the hypothesis that the effect of BCG prior to administration of subunit vaccine enhances BCG immunological memory (32). This finding is consistent with our study. FC was a better antigenic complex to the immune system compared to the results obtained from the PPE44: HSPX: FCG2a vaccine with the PPE44: HSPX protein: HIS, which stimulates a higher level of a cellular immune response. Some studies showed that targeting antigens to bind to the FC receptor at the cell surface may enhance their uptake and presentation to antigen donors T lymphocytes and modify the processing and supply process (33).

According to the studies by Probst et al. and Moradi et al., the use of FC fusion protein promotes the ability of both CD4+ and CD8+ T lymphocytes to elicit a protective immune response against intracellular bacteria is of great importance (34, 35).

The study has shown that upon the entry of the PPE44: HSPX complex, the fusion protein breaks apart under acidic conditions in the phagosome and becomes cytolytic. The fusion proteins covalently link at the hinge region by a disulfide bond, which enhances the immunogenic capacity of the antigens bound to the dimerization domain induced by Fc binding to the antigens the potential and the size of recombinant proteins, thereby reducing renal excretion. As a result, the half-life of recombinant proteins increases their lifespan (36-41)

Some studies showed that the binding of immunogenic viral proteins such as HIV and Ebola viruses to the second Fc region elicited a greater immune response against these viral infections (37-40). 

The Cytokine TNF-α response of experimental mice demonstrated the highest TNF-α release in the BCG immunized group, which was about 23% higher than the PHF group. Furthermore, IL-22 as an adjuvant in the vaccine formulation suppressed TNF-α release compared to the mere vaccine groups. Studies show that TNF-α is essential to control *M. tuberculosis* infection. It seems that a defect in TNF-α cytokine production resulted in uncontrolled infection (40). Studies show the potency of IL-22 cytokine as an adjuvant of a vaccine-inducing inflammatory cytokine such as IL-1β (41). Here, IL-22 in the vaccine formulation does not show a positive effect on the TNF-α as pro-inflammatory cytokine release, and it seems that cytokine suppressed pro-inflammatory cytokine release. This result is not in parallel with the result obtained in the previous work. This controversy may be due to antigen nature and the dose of IL-22 cytokine in the vaccine formulation.

Assessment of specific total IgG response shows that BCG was more potent than the PHF group in inducing humoral immune responses, and IL-22 in the vaccine formulation does not show a positive effect in the stimulation of humoral immune response in the PHF and BCG+PHF groups as compared with the mere vaccine. Also, this pattern was similar to specific IgG1 response for experimental groups, but IgG2a was increased in the PHF group adjuvanted with IL-22 compared to all experimental groups, while in the BCG group, adding IL-22 cytokine as the adjuvant does not show such positive effect in the induction of IgG1 response. Various studies show the beneficial effect of Il-22 cytokine as an adjuvant in combination with experimental vaccines in the induction of humoral immune responses (22, 40). Here in the TB vaccines model, we didn't achieve such a pattern for humoral immune response except for PHF, which indicated an increase in IgG1 level when IL-22 was added to the vaccine formulation. 

## Conclusion

**Overall, according to the potency of the vaccines to induce cellular and humoral immune responses, it seems that BCG, as the only licensed vaccine for TB infection, was more potent than the recombinant vaccine in inducing cellular and humoral immune responses and adding IL-22 in the vaccines formulations does not show a positive effect in the improvement of immune responses. Further studies are needed to find a more potent vaccine based on recombinant technology to be used instead of BCG in the human population.**

## Conflict of Interest

The authors declared no conflict of interest.

## Funding

None.
